# Obstetric Fistula in Burundi: a comprehensive approach to managing women with this neglected disease

**DOI:** 10.1186/1471-2393-13-164

**Published:** 2013-08-21

**Authors:** Katie Tayler-Smith, Rony Zachariah, Marcel Manzi, Wilma van den Boogaard, An Vandeborne, Aristide Bishinga, Eva De Plecker, Vincent Lambert, Bavo Christiaens, Gamaliel Sinabajije, Miguel Trelles, Stephan Goetghebuer, Tony Reid, Anthony Harries

**Affiliations:** 1 Médecins Sans Frontières, Medical department (Operational Research), Operational Center Brussels, MSF-Luxembourg, Luxembourg; 2 Médecins Sans Frontières, Bujumbura, Burundi; 3 Médecins Sans Frontières, Operational Centre Brussels, Brussels, Belgium; 4 Ministry of Health, Gitega, Burundi; 5 International Union against Tuberculosis and Lung Disease, Paris, France; 6 London School of Hygiene and Tropical Medicine, London, United Kingdom; 7 Medecins sans Frontieres (Operational center Brussels), Medical department (Operational research), Rue Dupré 94, 1090 Brussels, Belgium

**Keywords:** Obstetric fistula, Comprehensive management, Operational research, Burundi

## Abstract

**Background:**

In Burundi, the annual incidence of obstetric fistula is estimated to be 0.2-0.5% of all deliveries, with 1000–2000 new cases per year. Despite this relatively high incidence, national capacity for identifying and managing obstetric fistula is very limited. Thus, in July 2010, Medecins Sans Frontieres (MSF) set up a specialised Obstetric Fistula Centre in Gitega (Gitega Fistula Centre, GFC), the only permanent referral centre for obstetric fistula in Burundi. A comprehensive model of care is offered including psychosocial support, conservative and surgical management, post-operative care and follow-up. We describe this model of care, patient outcomes and the operational challenges.

**Methods:**

Descriptive study using routine programme data.

**Results:**

Between July 2010 and December 2011, 470 women with obstetric fistula presented for the first time at GFC, of whom 458 (98%) received treatment. Early urinary catheterization (conservative management) was successful in four out of 35 (11%) women. Of 454 (99%) women requiring surgical management, 394 (87%) were discharged with a closed fistula, of whom 301 (76%) were continent of urine and/or faeces, while 93 (24%) remained incontinent of urine and/or faeces. In 59 (13%) cases, the fistula was complex and could not be closed. Outcome status was unknown for one woman. Median duration of stay at GFC was 39 days (Interquartile range IQR, 31–51 days).

The main operational challenges included: i) early case finding and recruitment for conservative management, ii) national capacity building in obstetric fistula surgical repair, and iii) assessing the psychosocial impact of this model.

**Conclusion:**

In a rural African setting, it is feasible to implement a comprehensive package of fistula care using a dedicated fistula facility, and satisfactory surgical repair outcomes can be achieved. Several operational challenges are discussed.

## Background

While extremely rare in the developed world, obstetric fistula continues to be a common complication of childbirth in developing countries [1]. In most cases, obstetric fistula is a result of obstructed labour, occurring when the presenting part of the foetus cannot pass through the birth canal [2]. The most frequent cause of obstructed labour is cephalo-pelvic disproportion - a mismatch between the foetal head and the mother’s pelvic brim. The foetus may be too large in relation to the maternal pelvis or the pelvis may be malformed or underdeveloped [3]. Other causes of obstructed labour include mal-presentation or mal-position of the foetus rendering normal obstetrical mechanics impossible. Prolonged compression by the foetal head and subsequent necrosis of the soft tissues of the mothers’ vagina, bladder, urethra and/or rectum, and their subsequent sloughing away, leads to the formation of a hole (a fistula) between adjacent organs [4]. This often occurs between the vagina and bladder (vesicovaginal fistula, VVF), but can also develop between other organs, for example the vagina and rectum (rectovaginal fistula, RVF). In severe cases, more than one type of fistula can develop. The physical sequelae of obstetric fistula are urinary and/or faecal incontinence, which can lead to other medical complications such as infection, genital ulceration, pain and secondary infertility [4]. Obstetric fistula often has devastating psychosocial implications [5], in particular, from the accompanying smell that surrounds these women as a result of their urinary or faecal incontinence. This often leads to community ostracism and abandonment by husbands and families [1,6].

Obstetric fistulae occur in settings where access to obstetric care is limited [7] and, as such, tend to affect the most marginalised women in society. The social consequences of the condition contribute to further isolation [7]. International and national efforts to strengthen prevention and treatment of obstetric fistula are still lacking and the condition has rightly become regarded as a “neglected disease” [7-9].

In Burundi, in 2010, 40% of women still had no access to skilled attendants at birth and the caesarean section rate was estimated to be low at 4% [10] compared to the minimum acceptable caesarean rate of 5% [11]. Against this backdrop, the annual incidence of obstetric fistula has been estimated to be 0.2-0.5% of all deliveries, with 1000–2000 new cases per year [12]. Despite this relatively high incidence, national capacity for identifying and managing obstetric fistula is limited. Thus, in 2010, Medecins Sans Frontieres (MSF) set up a permanent referral centre for the management of women with obstetric fistula. Unlike the one-time surgical fistulae repair ‘camps’ that have become a common humanitarian venture in the last few decades, the MSF model of obstetric fistula care goes beyond just the technical act of surgical repair. It seeks to provide a comprehensive package of fistula care that promotes both physical and psychosocial recovery. While other programmes like MSF’s exist [13,14], there is limited published information, especially from rural Africa settings, describing the whole model of care offered together with the operational challenges and ways to address these.

Based on the MSF model of care for obstetric fistula in Burundi, we report on i) the package of comprehensive activities provided in a dedicated fistula facility, ii) conservative and surgical treatment outcomes, and iii) the operational challenges.

## Method

### Design

This was a descriptive study involving the retrospective review of routine programme data.

### Setting and population

Gitega Fistula Centre (GFC) was set up in July 2010 by MSF in collaboration with the Ministry of Health (MoH) and Handicap International. Located within the vicinity of Gitega Regional Hospital in Gitega town, GFC has dedicated infrastructure space but shares a number of the hospital’s departments including laundry, laboratory and radiology and has one of the hospital’s operating theatres designated entirely for fistula repair. GFC is the only permanent referral facility in Burundi to specialize in obstetric fistula management and it has the capacity to perform about 350–450 obstetric fistula surgical repairs per year. All care is offered free of charge.

This study included all women with an obstetric fistula seeking care for the first time at GFC between July 2010 and December 2011.

### Case finding

Women access obstetric fistula care at GFC in three ways: i) community screening sessions, ii) self referral to GFC, and iii) health centre referrals.

Community screening sessions take place twice a month in different provinces throughout Burundi. To encourage attendance at these sessions, extensive community awareness activities take place one month prior to each session. This includes intensive radio broadcasting one week prior to each session, with messages broadcasted three times a day across the three most popular radio stations in the target province. Women diagnosed with obstetric fistula during these screening sessions are given an appointment to return to the screening location about 1–2 weeks later, where MSF returns to transfer these women back to GFC free-of-charge.

To encourage self-referral and health centre referrals to GFC, MSF, in collaboration with Handicap International, has engaged in extensive community awareness activities on obstetric fistula and its treatment throughout Burundi, targeting and mobilising health facility staff and influential community members (religious leaders, village chiefs, local health promoters). Additionally, in April 2011, an OF telephone hotline was established at GFC to provide obstetric fistula-related information, advice and support (an average of 450 calls per month are received). Women calling with a suspected obstetric fistula are encouraged to come to GFC, with the offer of reimbursement of transport costs once they arrive at the centre. Woman unable to afford transport are urged to attend the next scheduled obstetric fistula screening session in their region, from where MSF can transport them back to GFC for free.

### Obstetric fistula diagnosis

All women with suspected obstetric fistula are examined by a GFC medical doctor. A simple digital pelvic examination is performed which involves palpating to feel for a fistula and if palpable, feeling for the size, location and associated problems such as vaginal scarring. If no fistula can be palpated, diagnosis is confirmed using dye and water tests. For a vesicovaginal fistula, this involves inserting a urinary catheter, filling the bladder with dye, clamping the catheter, and observing if the dye leaks through the vagina via the fistula. If the dye test is negative and urine leakage from the vagina is still clearly visible, further investigation is undertaken to better examine the entire urinary tract.

Detection of a rectovaginal fistula involves filling the vagina with water and the rectum with air and observing if air passes from the rectum, through the fistula, to form bubbles on the vaginal side of the passage.

Fistulae are classified according to the Kees Waaldijk classification system [15] (Table 1).

**Table 1:** **Classification of fistula according to type of surgery required based on their anatomic/physiologic location **[15]

**Fistula type or size**	**Sub-category**	**Description**
**Type I**		Fistula not involving the closing mechanism
**Type 11 A**		Fistula without (sub)total urethral involvement
	**a**	without circumferential defect*
	**b**	with circumferential defect
**Type 11 B**		Fistula with (sub)total urethral involvement
	**a**	without circumferential defect*
	**b**	with circumferential defect
**Type III**		Miscellaneous, e.g. ureteric and other exceptional fistula
**Small**		<2 cm
**Medium**		2**–**3 cm
**Large**		4**–**5 cm
**Extensive**		6 or more cm

* Circumferential defect: the complete separation of the urethra from the bladder.

### Obstetric fistula village at GFC

Women admitted to GFC stay in a fistula village which comprises four houses providing accommodation (up to 14 beds per house), shower and toilet facilities, an area for cooking and washing clothes, a kitchen, and a communal area for sharing meals and peer support activities. Woman can keep their children under the age of two years with them while staying in the village. If the village reaches capacity, three large tents are available to accommodate more women (additional capacity of 56 beds). Women at all stages of treatment (pre- and post-operatively) live together.

Four professional care takers look after the women in the village. All women receive various non-food items (including a blanket, a mosquito net, soap, toothpaste and toothbrush, and four sarongs) and women and children receive three free meals per day.

### Staffing resources and capacity building

GFC is staffed by one expatriate surgical expert in obstetric fistula repair (who is replaced by another usually every one to three months), a trainee expatriate surgeon, two national doctors, nine nurses, three social workers, four assistant social workers/nurses and four professional care takers. All staff are employed by MSF apart from three nurses who are employed by the MoH and who work entirely at GFC. A surgical training program for national staff is in place at GFC, with the aim of training at least one national doctor annually in the surgical management of obstetric fistula.

### Management of obstetric fistula

#### i) *Psychosocial support and education*

On admission, all women are assessed by a social worker in order to determine the psychosocial impact of obstetric fistula (e.g. rejection by her family/community; employment constraints and the financial repercussions of this etc.). Women undergo individual and group counseling and education on obstetric fistula throughout their stay. In addition, peer-support activities in the form of singing and animation activities take place in the communal area in the obstetric fistula village, in order to build solidarity and restore self-esteem among the women. Prior to discharge, women are counselled on how to ensure complete physical recovery, including family planning advice; the majority of women are discharged with a family planning method.

#### ii) *Conservative management*

Conservative treatment involves urinary catheterisation for four to six weeks. During the study period, the eligibility criteria for conservative treatment changed: initially they included any woman presenting with an obstetric fistula within six weeks of developing the fistula. However, based on a change in consensus, since June 2011 these criteria have included only women with a fistula present for three weeks or less and no larger than three cm in diameter. Health centre staff are encouraged to insert a catheter prior to referral to GFC if a new VVF is suspected following a recent delivery. Following catheter insertion, the fistula site is cleaned and the catheter changed once weekly at GFC. Fistula closure is assessed using the dye test. If fistula closure is not achieved after four to six week of conservative treatment, early surgical repair is scheduled.

#### iii) *Surgical management*

Women who are not eligible or who do not respond to conservative management are managed surgically. One operating theatre at the Gitega Regional Hospital is designated entirely for obstetric fistula surgical repair and all repairs are performed either by the expert surgeon or, under his supervision, the trainee doctors or surgeon. Following surgery, women are monitored for possible complications on a post-operative ward for three days before returning to the fistula village for three to four weeks. Patients are catheterised for two to four weeks post surgery (depending on the complexity of the fistula) at the end of which, fistula closure is assessed using the dye test. When the dye test is negative, the catheter is removed and the patient remains at GFC for a further three days. If the dye test is positive, catheterisation may be continued for another two weeks.

#### iv) Pelvic floor strengthening

Women are taught pelvic floor strengthening exercises (physiotherapy) to perform while at GFC (pre- and post-surgery) and after discharge, in order to enhance urinary continence.

#### v) Follow-up

Follow-up telephone calls by the social worker at GFC are scheduled for three and six months after discharge in order to assess continence and psychosocial status. If any continence problems are reported, a medical consultation back at GFC is scheduled. If any psychosocial problems are identified, then a community social worker is responsible for following this up. The psychosocial needs of the women are assessed and measures are then taken to try to address these needs. Where the required support is beyond the scope of the social worker, MSF collaborates with Handicap International and local associations for their inputs.

### Treatment outcomes

Treatment outcomes are determined just prior to discharge from GFC. The dye and water tests are done to determine fistula closure and the cough test to determine urinary continence (this involves asking the woman to cough and observing for any urine leakage). Treatment outcomes are classified as follows: closed fistula and no incontinence on cough test, closed fistula with persisting incontinence on cough test, or unclosed fistula. These outcomes are determined by the medical doctor.

### Management of other health related problems

With GFC being located within the complex of Gitega Regional Hospital, women with health related issues, aside from obstetric fistula, can access care and treatment for these.

### Data collection and statistical analysis

Patient data were sourced from individual patient records and registers kept at GFC and maintained and updated by the medical doctor in charge. These data were entered into an electronic database and were validated by cross-checking registers and patient records. The following variables were collected: date of presentation at GFC, age, marital status, parity, type of fistula (VVF, RVF or both), date of causal delivery, type of case (new or re-intervention), type of intervention (conservative or surgical), fistula closure status at discharge, and urine/faecal continence status at discharge. Data were analyzed using STATA/IC 8.0 software (Stata corporation, Texas 77845, USA).

### Ethics Statement

The study fulfilled the criteria for analysis of routine data by the MSF Ethics Review Board and the Burundi Ethics Committee, Bujumbura.

## Results

### Characteristics of the study population

Between July 2010 and December 2011, 470 women sought obstetric fistula care for the first time at GFC. Table 2 shows the demographic and obstetric characteristics of these women. Median age was 31 years (Interquartile range IQR, 25–40) and 407 (87%) women presented with a (VVF). The women had lived with their fistula for a median of six years prior to seeking treatment (Interquartile range,IQR 1–13 years).

**Table 2:** Demographic and obstetric characteristics of women with an obstetric fistula seeking care for the first time at GFC, Gitega, Burundi

***Variable***	**n (%)**
**Total**	470
**Age (years)**	
> 20	16 (3)
20 – 29	171 (36)
30 - 39	142 (30)
40 +	140 (30)
Unknown	1 (0.2)
Median, years (IQR)	31 (25–40)
**Marital status**	
Single	54 (11)
Married	255 (54)
Separated	105 (22)
Widowed	47 (10)
Unknown	9 (2)
**Parity**	
One birth	206 (44)
Two births	73 (15)
Three or more	181 (39)
Unknown	10 (2)
Median births (IQR)	2 (1–4)
**Type of obstetric fistula**	
VVF	407 (87)
RVF	42 (9)
VVF & RVF	21 (4)
**Duration of fistula (years)**	
< 1	109 (23)
1-4	83 (18)
5-10	89 (19)
10+	144 (31)
Unknown	45 (10)
Median, years (IQR)	6 (1–13)
**Initial Intervention at GFC**	
None*	12 (3)
Catheter only	4 (1)
Catheter followed by surgical repair	31 (7)
Surgical repair only	423 (90)

*GFC* Gitega fistula centre, *IQR* Interquartile range, *VVF* Vesico-vaginal fistula, *RVF* Recto-vaginal fistula.

* Several women did not receive treatment on their first admission to GFC due to the presence of other co-morbidities and/or complications.

Of the 470 women, 35 (7%) received conservative treatment and 423 (90%) received surgical repair only. Twelve women (3%) did not receive any treatment due to the presence of other co-morbidities and/or complications. Thirty one (89%) women receiving conservative treatment subsequently required surgical treatment due to an unsuccessful outcome with urinary catheterization (NB. 24 [77%] of these women did not fall under the June 2011 eligibility criteria for conservative management).

Median duration of stay at GFC was 39 days (Interquartile range, 31–51 days).

### Treatment outcomes

Of the four women whose fistula was closed using conservative treatment, all achieved urinary continence (all these women met the June 2011 eligibility criteria for conservative management – i.e. fistula present for ≤ 3 weeks and fistula ≤ 3 cm in diameter).

Table 3 shows the treatment outcomes for the 454 women who were managed surgically. Overall, 394 (87%) were discharged with a closed fistula, of whom 301 (76%) were continent of urine and/or faeces, while 93 (23%) remained incontinent despite fistula closure. In 59 (13%) cases, the fistula was complicated and could not be closed. A complex fistula was defined as including any of the following: Type II or III fistulae according to the Kees Waaldijk classification system [15]; multiple fistulae; RVF or mixed VVF/RVF; fistula > 4 cm; failed previous surgical repair attempts, (with the latter four based on the WHO classification of a complex fistula [16]). Women with a rectovaginal fistula (RVF) had better overall surgical outcomes compared to those with VVF as did women with a non-complex fistula. Outcome status was unknown for one women.

**Table 3:** Surgical outcomes at discharge for women receiving treatment at GFC, Gitega, Burundi according to the fistula type and the complexity of the fistula

Surgical outcome*	**Type of obstetric fistula**			**Complexity of the fistula**		**Total**
	**VVF**	**RVF**	**n (%) VVF &RVF n**	**Non-complex**	Complex**	
	**n (%)**	**n (%)**	**(%)**	**n (%)**	**n (%)**	
**Total**	**395**	**41**	**18**	**114**	**340**	**454**
**Fistula Closed**	**341 (86)**	**38 (93)**	**15 (83)**	**114 (100)**	**280 (82)**	**394 (87)**
Continent	258 (75)	36 (95)	7 (47)	99 (87)	202 (72)	301 (76)
Incontinent	83 (21)	2 (5)	8 (53)	15 (13)	78 (28)	93 (23)
**Fistula Unclosed**	**53 (13)**	**3 (7)**	**3 (17)**	**0 (0)**	**59 (17)**	**59 (13)**
**Fistula status unknown**	**1 (0.3)**	**0 (0)**	**0 (0)**	**0 (0)**	**1 (0.3)**	**1 (0.2)**

*GFC* Gitega fistula centre, *VVF* Vesico-vaginal fistula, *RVF* Recto-vaginal fistula.

* Includes the 31 women in whom conservative treatment was unsuccessful.

** Includes: multiple fistulae, RVF or mixed VVF/RVF, fistula greater than 4 cm in size, failed previous surgical repair attempts and/or type II or III fistula according to the Kees Waaldijk classification system [15].

## Discussion

This experience, over an 18-month period, shows how a comprehensive package of fistula care can be implemented using a dedicated facility in a rural African setting.

The strengths of this study are that: i) data come from a programme setting and thus are likely to reflect the operational reality on the ground, ii) the staff at GFC are well trained and supervised, and thus we feel that the clinical data are robust, and iii) a large number of women were included. The study limitations are that: i) follow-up data were incomplete and therefore it was not possible to report on longer term outcomes (such as the proportion of women with a closed fistula, but persisting incontinence, who eventually achieved continence; the incidence of complications - such as the breakdown of a closed fistula); ii) data were not available to assess the psychosocial and educational effects of this model of care, or how women perceived the quality of care that they received, or their social integration upon leaving GFC and iii) due to an absence of up-to-date estimates on the national incidence of obstetric fistula in Burundi, it was not possible to assess the coverage of our activities.

Despite these limitations, this experience of managing women with obstetric fistula in Burundi raises a number of important points for discussion.

First, in an era where obstetric fistula continues to be a neglected disease affecting a vulnerable, excluded and stigmatized population, a package of comprehensive care that seeks to address the multi-dimensional components of the condition seems essential. One time fistula camps that try to simply ‘fix the hole’ seem inadequate and may cause more harm than good in a number of ways: i) western surgeons may be ill-prepared to deal with the presentation of complex fistulae [17] - particularly when resources are scarce and surgical supplies unpredictable - and may lack the experience to know when operating is not in the best interest of the patient; ii) post-operative care and follow-up is often inadequate; and iii) the cultural norms and values of these women may be poorly understood and overlooked [18]. Our experience from Burundi demonstrates a possible way forward for managing women with obstetric fistula in rural Africa and corroborates the positive reports from other resource poor settings in Ethiopia and Nigeria, where similar models of care have been implemented [13,14]. In these programs, the surgeons performing the fistula repairs have extensive experience in this field, appropriate post-operative care and follow-up is ensured, and a holistic philosophy is adopted that emphasises care for the entire person, taking into account multi-system injuries and embracing the cultural norms of these women. At the time of this study, our programme was still in its infancy and had not yet developed the capacity to provide comprehensive physiotherapy services, or social and economic rehabilitation and reintegration. These services have since been developed at GFC and inclusion of such components in a fistula program would seem an important consideration.

Second, for the majority of women who had their obstetric fistula repaired surgically, fistula closure rates at the time of discharge were relatively good (87%) in comparison to reported rates of 73 -97% from other settings [19-22]. Nearly one in four women with successful obstetric fistula closure in our study, however, had residual incontinence. This is comparable with reports from other settings [21,22] and indicates that even after successful repair, incontinence can remain a significant problem. Such incontinence has been shown to improve over time (particularly if the fistula is of a less severe type), while in some cases, women who appear to be cured or have only mild incontinence at discharge develop recurrence or worsening of their symptoms [23]. Persisting incontinence may be related to detrusor overactivity, structural related reductions in bladder capacity and/or a deficiency in the closure mechanism of the bladder and urethra leading to urodynamic stress incontinence [24]. Urodynamic assessment to identify the specific reasons for persisting incontinence in women post obstetric fistula may provide clinically useful information. However, the feasibility of implementing such techniques and following up with appropriate solutions, may be difficult in resource-limited contexts like Burundi.

Third, with an absence of strong evidence on how best to deliver fistula care services [16], there are continuing discussions about whether stand-alone fistula centres or integrated hospital based fistula care services are more advantageous. General hospitals supporting fistula surgery are less costly and may be more accessible than stand-alone centres. However, issues around the availability of skilled staff and equipment to treat fistula cases (especially more complex cases), limited capacity to provide a holistic package of care and to accommodate the long-term stay of patients, are often challenges of this approach. The advantages of a centre like GFC range from the clinical expertise offered, the nurturing environment that is provided in the fistula village, the bringing together of women to share their suffering among peers, the exclusive use of an operating theatre and the opportunity for surgical training in fistula repair. The very presence of a dedicated centre may also help to raise community awareness about obstetric fistula. Furthermore, staff working at such centres, who develop a good understanding about the condition, may become powerful advocates for its prevention and treatment as has been the case with GFC. Finally, our experience of offering fistula care at GFC has allowed important lessons to be learnt about how to improve the approach and how to tailor it to the specific context of Burundi.

There were a number of operational challenges related to our experience. First, the wider issue of case finding and early recruitment was a major challenge, especially early detection of women with new fistulae for whom conservative treatment is often viable [25-27]. Half of the women who sought care in this study had lived with their fistula for five or more years, and of those women who underwent conservative management, nearly 90% had an unsuccessful outcome which was most likely linked to catheter insertion being too late. In women who have endured a prolonged obstructed labour, fistula formation can be prevented and small fistulae closed, by placement of an indwelling urinary catheter immediately post-delivery [25-27]. Implementing this preventive measure would require that: i) health care workers are trained appropriately, ii) urinary catheters are available at all delivery sites, iii) pregnant women are educated about the possible consequences of prolonged labour and obstetric fistula preventative measures (during antennal care visits at health centres and/or through traditional birth attendant (TBA) networks), iv) links with TBAs are established for the early referral of women who have had a prolonged labour, and v) monitoring and effective referral links are established when conservative management fails, including training of community workers to identify and refer women with obstetric fistula.

Second, transport costs were considered to be a major barrier to access to care for many women with obstetric fistula in Burundi. Thus, MSF offered transport cost re-imbursements and provided free transport where possible to GFC. The extent to which these measures covered all those women attempting to seek care at GFC is unclear however, and it is likely that we may have missed a proportion of women who might not have been able to afford the costs. In addition to transport costs, there may have been a proportion of women diagnosed with a fistula at the screening sessions, who never returned later to be taken by MSF to GFC. We would think that most women diagnosed with a fistula at these screening sessions, upon learning of the possibility of a cure at GFC, would be determined not to miss out on this opportunity. However, various barriers may have prevented this from being the case. It would thus be beneficial to evaluate attrition immediately after the screening stage and possible reasons for this.

Third, national capacity building in the required surgical procedures for obstetric fistula repair has been difficult. While the aim at GFC is to train one doctor annually in the surgical management of obstetric fistula, reaching this target has been a major challenge due to the lack of basic surgical skills among this cadre of healthcare worker. Our experience suggests that trainees need to have a minimum level of surgical skill, i.e. need to be surgeons or obstetricians/ gynaecologists, particularly when it comes to performing more complex surgical obstetric fistula repairs. While the ideal would be to recruit and train in-country specialists, if there is a general shortage of them, it would seem feasible to widen the pool of potential trainees to include volunteers from overseas who are prepared to make long-term commitments to working in medically deprived parts of the world like Burundi.

Fourth, access gaps of obstetric fistula activities in Burundi need to be better assessed. Ways forward include: i) mapping activities of other organisations involved in obstetric fistula, and ii) establishing up-to-date estimates of the incidence of obstetric fistula in Burundi in order to calculate the access gaps.

Fifth, while a large part of the MSF approach focussed on the provision of treatment for women suffering from fistula, we recognise that there needs to be much more emphasis on obstetric fistula prevention. This includes: i) better monitoring of labour (including measures to ensure that women have access to skilled birth attendance and the use of partograms to ensure that prolonged labour is identified and managed in a timely manner [28]; ii) improved access to emergency obstetric care (in particular swift and safe caesarean sections for women in obstructed labour). iii) competent medical care for women during and after obstructed labor, iv) improved access to family planning services, and v) increased education for girls and women [29]. We would advocate that if countries like Burundi want to reduce obstetric fistula, there must be political commitment towards making maternal health a priority and sustained investment into the strategies outlined above is needed. In the interim, sustained commitment is needed to ensure that women living with obstetric fistula have access to care and treatment. While the model of care proposed in this study is funded and supported by a non governmental organisation (NGO) – thus raising possible questions around sustainability - we would propose that it does not matter so much where the required funds come from (NGOs, Governments or donors), but that in all cases such investment needs to be committed in a sustained way. As progress in fistula prevention is made, and the incidence and prevalence of obstetric fistula starts to decline, a phasing out of fistula care services like GFC, could then be considered.

In conclusion, the MSF experience from Burundi demonstrates an encouraging way forward of how a comprehensive model of fistula care using a dedicated fistula facility can be implemented with satisfactory surgical treatment outcomes in a rural African setting. At the same time, we highlight some of the operational challenges that still need to be addressed.

## Competing interest

We have no conflict of interest to declare.

## Authors’ contribution

KTS, RZ, MM, AV, AB, EDP, VL, CB, GS, TR and ADH were involved with the conception and design. KTS wrote the study protocol which was substantially improved by RZ, MT, TR and ADH. KTS, MM, MT, AB, AV, WVDB and SG were involved with either project implementation or acquisition of study data. KTS did the initial analysis which was substantially improved by RZ. KTS and RZ wrote the first draft of the paper which was reviewed by all co-authors who all significantly contributed to the intellectual content. KTS handled the repeated revisions. All co-authors were involved with the final manuscript and approved the final paper.

## Pre-publication history

The pre-publication history for this paper can be accessed here:

http://www.biomedcentral.com/1471-2393/13/164/prepub
